# Tracking and predicting the treatment adherence of patients under rehabilitation: a three-wave longitudinal validation study for the Rehabilitation Adherence Inventory

**DOI:** 10.3389/fpsyg.2024.1284745

**Published:** 2024-04-12

**Authors:** Alfred S. Y. Lee, Shebe Siwei Xu, Patrick S. H. Yung, Michael T. Y. Ong, Chetwyn C. H. Chan, Joan S. K. Chung, Derwin K. C. Chan

**Affiliations:** ^1^Department of Rehabilitation Sciences, The Hong Kong Polytechnic University, Kowloon, Hong Kong SAR, China; ^2^Centre for Child and Family Science, The Education University of Hong Kong, New Territories, Hong Kong SAR, China; ^3^Department of Early Childhood Education, The Education University of Hong Kong, New Territories, Hong Kong SAR, China; ^4^Department of Orthopaedics and Traumatology, The Chinese University of Hong Kong, Shatin, Hong Kong SAR, China; ^5^Department of Psychology, The Education University of Hong Kong, New Territories, Hong Kong SAR, China

**Keywords:** rehabilitation adherence, self-determination theory, theory of planned behavior, ACL, sports medicine

## Abstract

This study aimed to develop and validate a new measurement tool, the Rehabilitation Adherence Inventory (RAI), to measure patients’ rehabilitation adherence. We recruited 236 patients with anterior cruciate ligament (ACL) ruptures from the United Kingdom (Mage = 33.58 ± 10.03, range = 18 to 59; female = 46.2%). Participants completed a survey, that measured their rehabilitation adherence, rehabilitation volume, psychological needs support, autonomous motivation, and intention at baseline, and at the 2nd and 4th month. Factorial, convergent, discriminant, concurrent, predictive, ecological validity and test–retest reliability of the RAI were tested via exploratory factor analysis (EFA), confirmatory factor analysis (CFA), and structural equation modelling (SEM). All the EFAs, CFAs, and SEMs yielded acceptable to excellent goodness-of-fit, χ2 = 10.51 to 224.12, df = 9 to 161, CFI > 0.95, TLI > 0.95, RMSEA <0.09 [90%C I < 0.06 to 0.12], SRMR <0.04. Results fully supported the RAI’s factorial, convergent, discriminant, and ecological validity, and test–retest reliability. The concurrent and predictive validity of the RAI was only partially supported because the RAI scores at baseline was positively associated with rehabilitation frequency at all time points (*r* = 0.34 to 0.38, *p* < 0.001), but its corresponding associations with rehabilitation duration were not statistically significant (*p* = 0.07 to 0.93). Overall, our findings suggest that this six-item RAI is a reliable and valid tool for evaluating patients’ rehabilitation adherence.

## Introduction

Rehabilitation programs serve the purpose of restoring muscle strength, joint mobility, and neuromuscular control, and facilitating patients’ return to their pre-injury activity levels. In the context of medical and rehabilitation settings, adherence refers to the extent to which individuals align their behavior with agreed-upon clinical recommendations (World Health Organization, 2003). It is crucial to emphasize the significance of treatment adherence for patients, as those who adhere to their prescribed treatments often experience more favourable recovery outcomes (Argent et al., 2018) and encounter fewer mental health issues, such as anxiety and depressive symptoms (Bachmann et al., 2018) than patients who do not adhere to their treatment. Nevertheless, low adherence to medical treatment or rehabilitation is often reported in various medical and rehabilitation contexts (Argent et al., 2018). For example, the dropout rate of patients receiving physiotherapy and exercise performance can reach 70% (Sluijs et al., 1993). The dropout rate of patients who were undertaking medical treatment following musculoskeletal injuries was up to 50% for those who were treated with medication (Lehane and McCarthy, 2007), and 50–65% for those who were prescribed home-based rehabilitation exercise (Bassett, 2003). High non-adherence rates were also reported in patients with chronic diseases; for example, 33% of diabetes patients in the residual cohort dropped out during each subsequent 6-month period (Graber et al., 1992). Poor treatment adherence affects the recovery progress of patients, resulting in economic burden on healthcare costs. Previous studies have highlighted the importance of assessing and monitoring of treatment adherence among patients during their rehabilitation process, especially for patients whose rehabilitation period is considerably long, such as patients who have undergone reconstruction surgery of the anterior cruciate ligament (Chan et al., 2017; Lee et al., 2020b).

To address the research gaps in the literature on the assessment of patients’ treatment adherence during their rehabilitation (Argent et al., 2018), we aimed to develop and validate the Rehabilitation Adherence Inventory (RAI) using a three-wave longitudinal study among patients with ACL rupture from the United Kingdom. We specifically chose ACL rupture as our focus because patients with this injury typically undergo extensive post-surgery rehabilitation, including strength and flexibility training, for a duration of six to 12 months. It is worth noting that low adherence to rehabilitation programs is frequently reported among ACL reconstruction patients due to the prolonged recovery process (Wright et al., 2014), which in turn may increase the risk of re-injury (Kaeding et al., 2015; Webster and Feller, 2016; Wiggins et al., 2016).

### Existing measures of patients’ rehabilitation adherence

Over the years, researchers have adopted different tools to measure rehabilitation adherence (Sluijs et al., 1993; Byerly et al., 1994). Apart from the rehabilitation attendance scores (Daly et al., 1995; Brewer et al., 2000, 2004; Shin et al., 2010), researchers have also used questionnaires on how much effort, commitment, or completion patients exert in their rehabilitation. The Sports Injury Rehabilitation Adherence Scale (SIRAS) (Brewer et al., 2000), the Hopkins Rehabilitation Engagement Rating Scale (HRERS) (Kortte et al., 2007), the Rehabilitation Adherence Questionnaire (RAQ) (Byerly et al., 1994), and the Self-Reported Sport Injury Rehabilitation Adherence Scale (SRSIRAS) (Chan et al., 2009; Lee et al., 2020b) appear to be more commonly used, or have received more evidence of validity than others. However, they are limited in terms of their usage and scale validity.

The SIRAS (Brewer et al., 2000) is a popular tool used to assess the rehabilitation adherence of patients with ACL rupture (Daly et al., 1995; Brewer et al., 2004). Although evidence supports the various types of scale validity of SIRAS, such as its internal consistency, test-rest reliability, and inter-rater reliability (Daly et al., 1995; Brewer et al., 2004), its usage is somewhat limited. This is because the SIRAS must be completed by rehabilitation professionals during clinic-based sessions, and thus cannot be completed by patients. On the other hand, the content validity, coverage of the items/dimensions, and factorial validity of SIRAS have not received solid supportive evidence (Shaw et al., 2005). Similarly, the HRERS (Kortte et al., 2007) is also one of the most commonly used scales of patients’ rehabilitation adherence (Mayhew et al., 2019). Although the HERES performs adequately in terms of factorial, convergent, discriminant and predictive validity (Mayhew et al., 2019), it is a therapist-reported scale that only focuses on the degree to which patients *engage* in their rehabilitation programmes from the perspective of their physiotherapists. As such, the responses of the SIRAS and HERES are more likely to reflect patients’ rehabilitation adherence during their clinic visits and are unlikely to capture the overall patients’ rehabilitation adherence, including their home-based rehabilitation.

The RAQ (Byerly et al., 1994) and the SRSIRAS (Chan et al., 2009; Lee et al., 2020b) are the two patient self-reported assessment tools often used in the literature (Byerly et al., 1994; Shin et al., 2010; Chan and Hagger, 2012). However, both scales have limitations regarding their usage or evidence of their validity. One critical issue with the RAQ is that the questionnaire length of 40 items increases the response burden of patients, and researchers might find it difficult to include the RAQ without notably extending the length of their questionnaire. In addition, its score reliability and validity have been reported to be either inadequate or poor (Brewer et al., 1999). For instance, Brewer et al. (1999) reported that the psychometric properties of the RAQ were weak, with the subscales failing to exhibit adequate criterion validity or internal consistency. Although the RAQ was later modified and redeveloped to become the Modified Rehabilitation Adherence Questionnaire (M-RAQ) (Shin et al., 2010; McLean et al., 2017), a full spectrum of validity was not tested in the study, and the convergent validity remains poor.

The SRSIRAS, on the other hand (Chan et al., 2009; Lee et al., 2020b) only includes two items, so completing it is unlikely to induce a response burden. In addition, the convergent validity of the SRSIRAS has been supported by acceptable Cronbach’s alphas in previous studies, and its concurrent validity is supported by significant correlations between the SRSIRAS scores and autonomous motivation and intention (Chan et al., 2017; Lee et al., 2020b; Chan et al., 2020c). However, because it only contains two items, the coverage of item content may be limited, and factor analysis cannot be properly performed. As such, the evidence regarding its content validity and factorial validity is limited.

In summary, existing evidence in the literature can only provide some support regarding the convergent validity of the measurement tools used to assess patient rehabilitation adherence. However, few tools have been developed for patient self-assessment, and the development of these tools has not undergone rigorous examinations of the scale validity and reliability. Indeed, a full spectrum of validity is important for psychometric testing. These indices include:

- factorial validity (i.e., whether the factor structure of the measurement tool is logical and robust);
- convergent validity (i.e., whether the measurement tool can correlate with the scores of similar scales or constructs);
- discriminant validity (i.e., the measurement tool evaluates a unique construct whose variance is independent from that of other factors);
- test–retest reliability (i.e., the extent to which the scores of the measurement tool are consistent over time);
- concurrent validity (i.e., the extent to which the measurement tool can explain the variance of certain outcome variables);
- predictive validity (i.e., the extent to which the measurement tool is predictive to future outcomes);
- ecological validity [i.e., the extent to which the scores of the measurement tool behave logically in the clinical setting and are responsive to social environmental factors (Hagger and Chatzisarantis, 2009; Chan et al., 2019, 2020a)].

### The present study

The objective of the present study was to develop and validate a new measurement tool called the Rehabilitation Adherence Inventory (RAI). This tool was specifically designed to assess patients’ adherence to rehabilitation protocols. To achieve this goal, a three-wave longitudinal study design was employed, allowing for a comprehensive evaluation of adherence over time. We hypothesise that the following validity indices would be exhibited as follows:

*(H1) Factorial validity*: consistent with the factor structure of the SRSIRAS (Chan et al., 2009; Lee et al., 2020b), the items of the RAI would load on one factor, *rehabilitation adherence*. The one-factor model would yield acceptable goodness-of-fit. The score reliability of this factor would be acceptable.
*(H2) Convergent validity*: The latent factor of RAI would be positively related to the scores of rehabilitation adherence assessed by the Self-Reported Sport Injury Rehabilitation Adherence Scale (SRSIRAS) (Chan et al., 2009; Lee et al., 2020b), and also psychological factors that were shown to correlate with indicators of rehabilitation adherence, including autonomous motivation and intention (Chan et al., 2017; Lee et al., 2020b; Chan et al., 2020c). Autonomous motivation toward rehabilitation is defined as an individual’s intrinsic drive and personal commitment to engaging in and adhering to their rehabilitation activities (Chan et al., 2009), while intention to rehabilitation refers to an individual’s conscious decision and planned course of action to engage in and pursue rehabilitation activities and treatment plans (Lee et al., 2020b). Previous studies have indicated that autonomous motivation and intention are significant predictors of behavioral adherence (Chan et al., 2017; Goddard et al., 2020; Lee et al., 2020a,b).
*(H3) Discriminant validity*: the variance of the RAI factor would be higher than its shared variance with autonomous motivation and intention, but not with the scores of rehabilitation adherence assessed by the SRSIRAS.
*(H4) Test-retest reliability*: the RAI factor at baseline, and the 2nd and 4th month would be positively correlated with each other.
*(H5) Concurrent validity*: the RAI factor would be positively associated with rehabilitation volume (i.e., *frequency and duration of rehabilitation*) within each time point of assessment.
*(H6) Predictive validity*: RAI factor at baseline would be positively associated with the future (i.e., 2nd and 4th month) rehabilitation volume (i.e., *frequency and duration of rehabilitation*).
*(H7) Ecological validity*: the scores of RAI factor would decline over time (H7a), but may increase when patients perceived higher psychological need support from their physiotherapists (H7b). Previous studies investigating the motivation and rehabilitation adherence among patients who underwent reconstruction surgery of ACL reported these behavioral patterns (Chan et al., 2009; Lee et al., 2020b).

## Method

### Scale development

The study received ethical approval from the Human Research Ethics Committee of the first author’s institution (blinded for review). The RAI aims to conceptualise patients’ adherence toward the rehabilitation programme. The development of the RAI was based on the results of qualitative interviews with four patients who had suffered from ACL rupture, and the assessment tools of behavioral adherence among ACL patients (Chan et al., 2009; Lee et al., 2020b, 2021), and other types of patients who underwent rehabilitation (Brewer, 1998). The initial nine items were developed and sent out for review. These items take patient commitment to follow rehabilitation, such as their effort, frequency and completion, into account. The reviewer team comprises two orthopaedic surgeons, two physiotherapists, and two behavioral medicine researchers, and one psychologist who are experienced experts and have publication records in the field of sport medicine and rehabilitation sciences. They reviewed the face validity of the items by providing feedback on the content relevance, coverage, and clarity of the items (Chan et al., 2019). After multiple exchanges, the team reduced three items from the initial pool of items due to redundancy. The single-factor six-item RAI is displayed in Appendix A.

### Participants and procedures

The invitation was sent out through Prolific, a participant recruitment platform targeting individuals in the United Kingdom from July 2021 to August 2021. Individuals were eligible for inclusion in the study if they met the following criteria:

1. were adults aged between 18 and 60 years
2. had been diagnosed with ACL rupture
3. had undergone ACL reconstruction within the last 12 months

A total of 2031 individuals were assessed for eligibility. Informed consent was obtained from the individuals (*N* = 287) who met the inclusion criteria. Finally, 236 patients with ACL rupture (*M*_age_ = 33.58 ± 10.03, range = 18 to 59; female = 46.2%) agreed to participate in the current study. Participants were asked to complete a survey package that measured the psychological variables related to behavioral adherence (e.g., psychological need support, autonomous motivation and intention) at baseline, and at the 2nd, and 4th month after the baseline. On average, participants had ruptured their ACL 9.43 (*SD* = 5.03) months ago and received the ACL reconstruction surgery 6.61 (SD = 3.39) months before baseline assessment. According to the baseline assessments, 83 participants (35.2%) reported they suffered from meniscus injuries alongside ACL rupture.

### Measures

#### Rehabilitation adherence

The six-item RAI was used to measure participants’ rehabilitation adherence. Participants rated the items, such as “I fully commit to the rehabilitation program,” on a seven-point Likert scale ranging from 1 (*Strongly disagree*) to 7 (*Strongly Agree*). The 7-point Likert scale was adopted because studies have supported the use of a 7-point Likert scale rather than other options, such as a 5-point Likert scale (Finstad, 2010; Taherdoost, 2019). Secondly, we employed a previously validated adherence scale, the SRSIRAS (Chan et al., 2009; Lee et al., 2020b), to comprehensively validate the RAI. This scale measures participants’ *frequency* (i.e., “How frequently do you follow your prescribed rehabilitation program?”) and *effort* (i.e., “How much effort do you put on completing your prescribed rehabilitation program?”) in completing rehabilitation. Participants rated the items on seven-point Likert scales (1 = *Never/Minimum effort* and 7 = *Often/Maximum effort*). The scale has demonstrated good internal consistency (Cronbach’s alpha = 0.87) (Lee et al., 2020b).

#### Rehabilitation volume

We measured participants’ rehabilitation volume by assessing their *frequency of rehabilitation* (“During the last 7 days, how many days did you follow the rehabilitation program?”) and *duration of rehabilitation* (“In a typical day of the last 7 days, how much time did you spend in the rehabilitation program?”) in the past week.

#### Psychological needs support

The six-item Health Care Climate Questionnaire (HCCQ) (Williams et al., 1996) was used to assess participants’ perceived psychological needs support from their physiotherapists on a seven-point Likert scale (1 = *Not at all true* and 7 = *Very true*). A sample item is “My physiotherapist listens to how I would like to do things.” The scale showed good reliability (i.e., α = 0.95) in a previous study (Lee et al., 2020b).

#### Autonomous motivation

We adopted the five-item Treatment Self-Regulation Questionnaire (TSRQ) (Levesque et al., 2006) to measure participants’ autonomous motivation in rehabilitation exercises. Participants rated the items (e.g., “It is important to me that my efforts succeed”) on a seven-point Likert scale from 1 (*Not at all true*) to 7 (*Very true*). The scale was suggested to be reliable (i.e., Ω = 0.78) in a previous study (Lee et al., 2023).

#### Intention

We measured participants’ intentions (e.g., “I plan to engage in all the recommended rehabilitation activities in the forthcoming month”) toward their compliance with the rehabilitation program using the three-item injury rehabilitation version of the theory of planned behavior scale (Lee et al., 2020b). Participants rated the items on a seven-point Likert scale (1 = *Strongly disagree* and 7 = *Strongly agree*). The scale exhibited good internal consistency (i.e., Ω = 0.98) in a previous study (Lee et al., 2023).

### Data analysis

To examine the factorial validity of the RAI (H1), we adopted exploratory factor analysis (EFA) with oblique goemin rotation on the baseline, 2nd and 4th month data. Cronbach’s alpha coefficients and inter-item correlations for the RAI factor were computed for factorial validity (H1). The convergent (H2) and discriminant (H3) validity of the RAI were examined using the baseline data. For convergent validity (H2), we estimated the correlations between the RAI factor and autonomous motivation, intention and the SRSIRAS variable in a confirmatory factor analysis (CFA) model. For discriminant validity (H3), we estimated the shared variance between the factors and the average variance extracted (AVE) for each factor, using the same CFA model. When the AVE exceeds the shared variance, discriminant validity is supported. The SRSIRAS variable was included as an observed variable because the scale has only two items.

We used the baseline, 2nd and 4th month data to investigate the test–retest reliability (H4), and concurrent (H5), predictive (H6) and ecological (H7) validity of the RAI. For the test–retest reliability (H4), we examined the correlation between RAI factor across baseline, and the 2nd and 4th month in CFA. For concurrent validity (H5), using CFA, we estimated correlations between the RAI factor and rehabilitation volume (i.e., the *frequency* and *duration* of rehabilitation) at baseline, and the 2nd and 4th month assessment. For the predictive validity (H6), CFA evaluated the factor correlations between the baseline RAI scores and the rehabilitation volume at the 2nd and 4th month. We used two approaches to test the ecological validity (H7) of the RAI. Three paired-samples *t*-tests (i.e., baseline vs. 2nd month, baseline vs. 4th month and 2nd month vs. 4th month) were conducted to examine if the RAI scores declined significantly over the three time points (H7a). Secondly, to examine H7b, we developed two models to predict perspective RAI scores using psychological need support (i.e., baseline psychological need support → 2nd month RAI and 2nd month psychological need support → 4th month RAI) via structural equation modelling (SEM).

We conducted the above analysis using Mplus version 8.4 (Muthén and Muthén, 2017). The conventional fit indices were adopted to assess the model fit, including the Comparative fit index (CFI), the Tucker-Lewis Index (TLI), the root mean square error of approximation (RMSEA), and the standardised root mean square residual (SRMR). Models were considered to have acceptable goodness-of-fit with the data if the CFI and TLI values approached or exceeded 0.90 and the RMSEA and SRMR values were below 0.08 (Hu and Bentler, 1999). Regarding missing data, 48 and 79 participants did not complete the 2nd and 4th month assessment, respectively, yielding retention rates of 20.33 and 33.48%, which were indeed consistent with typical longitudinal study attrition ranges (Gustavson et al., 2012). Data were missing completely at random (MCAR) based on the results of the Little’s MCAR (*χ*^2^ = 58.89, *df* = 72, *p* = 0.87) (Little and Rubin, 2019). We employed maximum likelihood with robust standard errors (MLR) estimation, which adjusts the likelihood function in that each case contributes information to the variables, to handle missing data in our analyses (Muthén and Muthén, 2017). The datasets generated during and/or analysed during the current study are available from the corresponding author upon request.

## Results

### Scale validation

For the factorial validity (H1), the three sets of EFAs yielded adequate goodness-of-fit for the single-factor model, *χ*^2^ = 10.51 to 22.25, *df* = 9, CFI > 0.98, TLI > 0.97, RMSEA <0.09 [90% CI < 0.04 to 0.14], SRMR <0.03. The fit indices did not improve for the two-factor model at either baseline or 4th month models. The factor loadings of the six items of the RAI ranged from 0.67 to 0.88, *p* < 0.001. The inter-item correlations ranged from 0.48 to 0.72, *p* < 0.001, yielding a Cronbach’s alpha coefficient of 0.90, supporting the factorial validity (H1).

The CFA model that tested the convergent (H2) and discriminant (H3) validity displayed excellent goodness-of-fit, χ^2^ = 182.76, *df* = 161, CFI = 0.99, TLI = 0.99, RMSEA = 0.02 [90% CI = 0.00 to 0.04], SRMR = 0.04. The latent RAI factor was positively associated with autonomous motivation (*r* = 0.63, *p* < 0.001), intention (*r* = 0.74, *p* < 0.001) and the SRSIRAS variable (*r* = 0.81, *p* < 0.001). The AVES of the RAI factor (i.e., AVE = 0.62) were higher than the share variance with the study variables (range = 0.40 to 0.55), except for the SRSIRAS variable (i.e., 0.65). The results aligned with our H2 and H3. See Table 1.

**Table 1:** Correlation matrix, distribution, and validity indices of the study variables at baseline.

	1	2	3	4	5	6
1. RAI	1					
2. Autonomous motivation	0.63^***^	1				
3. Intention	0.74^***^	0.74^***^	1			
4. SRIRAS	0.81^***^	0.54^***^	0.74^***^	1		
5. Rehabilitation frequency	0.38^***^	0.54^***^	0.50^***^	0.54^***^	1	
6. Rehabilitation duration	0.09	−0.02	0.05	0.08	0.09	1
Mean	5.89	5.98	6.12	5.85	5.09	52.83
SD	0.88	0.90	0.95	0.87	1.68	45.62
Cronbach’s alpha	0.90	0.75	0.90	0.78	N/A	N/A
AVE	0.62	0.43	0.75	N/A	N/A	N/A
Range of shared variance	0.40–0.65	0.30–0.58	0.54–0.59	0.30–0.65	0.01–0.29	0.00–0.01
Range of factor loading	0.67–0.84	0.52–0.79	0.85–0.89	N/A	N/A	N/A

RAI, Rehabilitation Adherence Inventory; SRIRAS, self-reported injury rehabilitation adherence scale; AVE, average variance extracted; N/A, not applicable. ^***^p < 0.001.

For the test–retest validity (H4), the CFA model showed adequate goodness-of-fit, χ^2^ = 224.12, *df* = 132, CFI = 0.95, TLI = 0.95, RMSEA = 0.05 [90% CI = 0.04 to 0.07], SRMR = 0.05. The RAI latent factors at baseline, and at the 2nd and 4th month assessments were positively correlated (*r* = 0.71 to 0.78, *p* < 0.001), supporting our H4.

In terms of concurrent validity (H5), the three CFA models yielded adequate goodness-of-fit, χ^2^ = 24.68 to 47.90, *df* = 20, CFI > 0.96, TLI > 0.95, RMSEA <0.08 [90% CI < 0.06 to 0.12], SRMR <0.04. The RAI factors were positively correlated with the *rehabilitation frequency* at each time point of assessment (*r* = 0.34 to 0.38, *p* < 0.001). Yet, we did not find a significant association between RAI factors and *rehabilitation duration* in any of the three models (*r* = 0.01 to 0.08, *p* = 0.28 to 0.93). Our H5 was only partially supported.

For the predictive validity (H6), the CFA model had excellent goodness-of-fit with the data, χ^2^ = 38.67, *df* = 29, CFI = 0.99, TLI = 0.98, RMSEA = 0.04 [90% CI = 0.00 to 0.07], SRMR = 0.03. The baseline RAI latent factor was positively associated with the *rehabilitation frequency* at the 2nd (*r* = 0.33, *p* < 0.001) and 4th month (*r* = 0.39, *p* < 0.001). However, there was no significant correlation between baseline RAI latent factor with *rehabilitation duration* in 2nd (*r* = 0.04, *p* = 0.57) and 4th month (*r* = 0.12, *p* = 0.07), partially supporting our H6.

For the ecological validity (H7a), the paired-samples *t*-test revealed that the RAI scores declined significantly from baseline to the 2nd month (*t* = 3.09, *p* = 0.01) and 4th month (*t* = 3.75, *p* < 0.001) but not between the 2nd month and 4th month (*t* = 0.89, *p* = 0.38), consistent with H7a to a large extent. Secondly, the two SDT SEMs showed adequate goodness-of-fit, χ^2^ = 76.63 to 104.52, *df* = 53, CFI > 0.96, TLI > 0.95, RMSEA <0.06 [90% CI < 0.05 to 0.08], SRMR <0.04. The psychological need support significantly predicted the future RAI factors (*β* = 0.32 to 0.33, *p* < 0.001; H7b), supporting our H7b.

## Discussion

Patients who adhere to treatment tend to have more favourable recovery outcomes and better psychological well-being than those who do not (Argent et al., 2018; Bachmann et al., 2018). To precisely assess and monitor patients’ treatment adherence during their rehabilitation, it is essential to develop an accurate, reliable and validated measure. This study was conducted to develop and validate a novel tool, known as the Rehabilitation Adherence Inventory (RAI), to document patients’ rehabilitation adherence. Results from 236 patients with ACL rupture in the UK supported the factorial (H1), convergent (H2), discriminant (H3), test–retest reliability (H4), and ecological validity (H7) of the RAI. The concurrent (H5) and predictive (H6) validity of the RAI were partially supported because the RAI factor was predictive to rehabilitation frequency but not to rehabilitation duration. Overall, the patterns of results in our three-wave longitudinal validation study generally supported the full spectrum of reliability and validity of the RAI.

### Advancement of the RAI

The RAI is presented as a scale of rehabilitation adherence that measures patients’ perspectives. This may address the limitations of previous studies that only assessed patients’ adherence from the perspectives of the medical professionals (McLean et al., 2017). As such, the introduction of the RAI aligns with the principles behind patient-centred outcome assessment, whereby medical professionals view patients as their collaborative partners when guiding the rehabilitation process (Hewlett, 2003). The length of the RAI appears to take a good balance between content coverage (as reflected by the responses from orthopaedic surgeons and physiotherapists) and the ease of response burden (as reflected by the quantitative findings from the three-wave patient dataset).

Compared to existing scales of rehabilitation adherence, the RAI has achieved a very high standard of reliability and validity when. This is perhaps because there has not been any self-reported rehabilitation adherence scale that can fulfil the criteria of such a comprehensive set of validity assessments, such as test–retest reliability, discriminant validity, predictive validity, and ecological validity, recommended by the literature (Chan et al., 2020a). The positive associations between RAI scores, autonomous motivation, and intention not only provided evidence for supporting the convergent validity of the scale, but it also may show that ACL patients with higher rehabilitation adherence were more likely to have higher intrinsic values and long-term prescribed commitment to their rehabilitation programmes. Similarly, the positive associations between psychological need support and the future RAI scores not only provided evidence for the ecological validity, but they also showed that ACL patients had better rehabilitation adherence when they perceived that their physiotherapists supported their psychological needs. Such findings are in congruent with that of the previous studies conducted among patients with ACL rupture (Chan et al., 2009; Lee et al., 2020b) or other medical settings (Murray et al., 2015), thus the RAI scores appear to be reflective of the key motivational and behavioral factors of patients’ rehabilitation identified in the literature.

In summary, the generally positive results indicate that the RAI provides a robust and patient-centred assessment of rehabilitation adherence for both clinical and home-based rehabilitation settings. Although our study tested the validity of the RAI among patients with ACL rupture only, the generic item content of the scale could make it applicable to evaluating or monitoring the rehabilitation adherence of patients in other clinical settings, such as those with strokes, spinal cord injury, and other musculoskeletal injuries (Goddard et al., 2020). We hope future studies can apply and validate the RAI in diverse clinical settings to enhance the generalisability of the RAI usage.

### Some noteworthy observations

To examine whether the rehabilitation adherence measures explain explanatory to patients’ rehabilitation behaviors, former studies have mostly used rehabilitation attendance at clinics as the primary outcome variable (Daly et al., 1995; Brewer et al., 2000, 2004; Shin et al., 2010), but the cross-sectional designs of these studies were unable to provide evidence regarding the predictive power of the rehabilitation adherence measures. As compared to these previous studies, the RAI may provide more robust evidence of the concurrent validity and predictive power of the rehabilitation adherence measure because our study adopted a three-wave longitudinal design and rehabilitation volume (i.e., rehabilitation *frequency* and *duration*) as the concurrent variable that may reflect either home-based or clinic-based rehabilitation. However, it is interesting to note that RAI scores can only explain the *rehabilitation frequency* but not the *rehabilitation duration* of patients. One plausible reason is that the recommended duration of rehabilitation exercise may vary alongside the rehabilitation period, and is subject to the recovery progress of the patients and the prescription of medical professionals (Grant, 2013; Filbay and Grindem, 2019). Therefore, the rehabilitation duration may not always reflect the extent to which patients comply with the prescribed rehabilitation programmes.

This argument is consonant with the changing pattern of the RAI scores in our study. The RAI scores decreased significantly from baseline to the 2nd month and 4th month of assessment; however, the decline was not significant between the 2nd month and 4th month of assessment. The variation of the RAI scores in our study may delineate the pattern of rehabilitation along with the stages of recovery, whereby patients are generally more committed to their treatment during the initial stages than the final stages (Schultz, 2015). The pattern of change we observed not only provides support to the ecological validity of RAI, but may also underscore the heightened risk of patients’ non-compliance or dropout in the final stages of treatment. Promotion and monitoring of patients’ adherence at this stage of treatment, for instance, by the use of adaptive motivational patterns (Chan and Hagger, 2012; Lee et al., 2020b) and motivational interviewing (Medley and Powell, 2010), could be particularly important for the prevention of relapse or re-injury of the medical conditions.

## Limitations

To the best of our knowledge, the RAI is the first patient-reported adherence scale that has been subjected to a comprehensive testing of validity. Despite the notable strengths of our study, we must point out a few study limitations and discuss how further studies can address these limitations. First, we did not include any clinical measures of recovery outcomes, so the evidence of concurrent validity, differential validity, and the clinical significance of the RAI could be impaired and warrants further studies to supplement. Second, RAI is a self-reported measure, so the responses are subject to recall bias, social desirability, and other method artefacts (Chan et al., 2020b). Future studies should examine the inter-rater reliability of the RAI by comparing the self-reported and other-reported scores of the RAI. Third, the RAI aims to capture patients’ overall treatment adherence without specifying if it refers to clinic-based or home-based treatment activities. To enhance the applicability of the RAI in diverse therapeutic contexts, future research should focus on examining the validity and reliability of the RAI, specifically within clinic-based and home-based treatment settings (Frost et al., 2017). Fourth, in the present study, participants’ demographic characteristics were limited to age, sex, and the medical history of ACL rupture. To provide a more comprehensive understanding, future studies could consider incorporating additional crucial demographic information, such as education, occupation, and socioeconomic status. Fifth, the participants included in the present study with ACL rupture were drawn from diverse backgrounds, including both athletes and non-athletes within the general public. The heterogeneity of the background of our sample was advantageous because it served the primary objective of this study, that was, to evaluate the suitability of the RAI for all individuals undergoing rehabilitation after experiencing an ACL rupture. However, it is worth noting that individuals with different backgrounds (e.g., occupations, sport levels) may have different perspectives and interpretations of the RAI items. Formally examining or comparing the psychometric properties of the RAI between individuals with different occupations (e.g., athletes versus non-athletes) will provide a more comprehensive evaluation on the validity of the RAI in diverse populations. Finally, we only examined RAI among a homogenous type of patient from the UK who underwent reconstruction surgery following ACL ruptures, so the generalisability of our findings could be restricted. Future studies should further examine the RAI with diverse patient samples with various medical conditions and cultural backgrounds.

## Conclusion

Documenting participant adherence is necessary to monitor patient progress and to help determine if the improvement is attributable to non−/adherence or ineffectiveness of prescribed therapies. The Rehabilitation Adherence Inventory (RAI) was developed and validated to precisely measure and monitor patient adherence. Using the three-wave longitudinal data collected in the UK, we provided preliminary support for the factorial validity, convergent validity, discriminant validity, test–retest reliability, concurrent validity, predictive validity, and ecological validity of the six-item RAI. Based on the current results, the RAI performs well as a valuable tool for measuring adherence as part of the rehabilitation process. The scale should be validated in other patient populations and cultural groups to further demonstrate its validity.

## Data availability statement

The datasets generated during and/or analysed during the current study are available from the corresponding author upon request.

## Ethics statement

The studies involving humans were approved by Human Research Ethics Committee of The Education University of Hong Kong. The studies were conducted in accordance with the local legislation and institutional requirements. The participants provided their written informed consent to participate in this study.

## Author contributions

AL: Conceptualization, Data curation, Formal analysis, Investigation, Methodology, Project administration, Software, Validation, Visualization, Writing – original draft, Writing – review & editing. SX: Investigation, Writing – original draft, Writing – review & editing, Conceptualization, Data curation, Formal analysis, Validation. PY: Conceptualization, Project administration, Resources, Supervision, Writing – review & editing, Investigation, Methodology, Validation. MO: Investigation, Project administration, Resources, Supervision, Writing – review & editing, Conceptualization, Methodology, Validation. CC: Conceptualization, Project administration, Supervision, Validation, Visualization, Writing – review & editing, Formal analysis. JC: Investigation, Validation, Writing – review & editing, Conceptualization, Data curation, Formal analysis. DC: Conceptualization, Data curation, Formal analysis, Funding acquisition, Investigation, Methodology, Project administration, Resources, Supervision, Validation, Visualization, Writing – review & editing, Software.

## Appendix A

Rehabilitation adherence inventory.

1. I put lots of effort into completing my rehabilitation program.
2. I work very hard to do the exercises in my rehabilitation program.
3. I try my best in following my rehabilitation program.
4. I frequently work on my rehabilitation program.
5. I fully commit to the rehabilitation program.
6. Nothing will stop me from following my rehabilitation program.
